# Chronic rhinosinusitis: an under-researched epidemic

**DOI:** 10.1186/s40463-015-0064-8

**Published:** 2015-03-05

**Authors:** Luke Rudmik

**Affiliations:** Division of Otolaryngology – Head and Neck Surgery, Department of Surgery, University of Calgary, Richmond Road Diagnostic and Treatment Centre, 1820 Richmond Rd., SW, T2T 5C7 Calgary, Alberta Canada

**Keywords:** Chronic sinusitis, Rhinosinusitis, Burden of disease, Research prioritization, Publication volume

## Abstract

**Background:**

Chronic rhinosinusitis (CRS) is a highly prevalent inflammatory disease with significant impacts on patient quality of life and daily productivity. Evaluating the volume of research on CRS, relative to similar chronic diseases, may provide insight into current disparities in research prioritization.

**Methods:**

A systematic review was performed using Ovid MEDLINE (R) (1970 – December 31st, 2014) to define the volume of research publications for CRS, asthma, and diabetes mellitus (DM). Primary outcomes were overall volume of research publications and volume of publications per year. A subgroup analysis was performed using chi-square (χ2) omnibus test with 2×3 contingency tables to identify significant differences in the proportion of total randomized controlled trials, systematic reviews, meta-analyses, and economic evaluation publications between CRS, asthma, and DM groups.

**Results:**

There were substantial disparities in the volume of research published over the last 45 years for CRS (n = 7,962), asthma (n = 136,652), and DM (n = 337,411). Although the volume of research for CRS in increasing, the disparities in the annual publication volumes between CRS, asthma, and DM appeared consistent over the last 45 years.

**Conclusions:**

Outcomes from this review have demonstrated a large disparity in the volume of published research for CRS compared to asthma and DM. Given the similarities in prevalence rates, impact on quality of life and economic burden, the relative under supply of CRS research should prompt efforts to increase research prioritization for this chronic disease.

## Background

The advancement of medical knowledge with subsequent translation into real world improvements is a key mandate for several stakeholders in health care [1-3]. Peer-reviewed publication is an essential vehicle to share newly acquired knowledge, and without publication of high quality research, advancements in the quality of care will be limited. Since there are scarce resources to support medical research, it is important to prioritize resources toward the highest impact diseases, however, due to the large number of different stakeholders involved in health care, research prioritization is a very complex process [4]. Several factors influence research priority setting, however, one important factor is the estimated burden of disease [5-7].

Chronic rhinosinusitis (CRS) not only represents one of the most prevalent chronic diseases in developed countries [8] but also represents a substantial burden of disease on patient quality of life [9], productivity [10], and health care spending [11]. Based on the best available evidence, the prevalence of CRS in North America and Europe likely falls within the range of 5% and 12% [12-14] which rivals and potentially exceeds that of other common chronic diseases such as asthma and diabetes mellitus (DM) (Table 1) [12,15-18]. If research prioritization accurately reflects burden of disease, then the volume and quality of publications should be somewhat similar between diseases with similar burden of disease.

**Table 1:** **Geographic prevalence rates per chronic disease**

**Chronic disease**	**Prevalence**			
	**Canada**	**US**	**Europe/UK**	**Mean**
**CRS**	5.7% [13]	12.1% [12]	10.9% [14]	9.6%
**Asthma**	7.9% [15]	8% [12]	3.8% [16]	6.6%
**DM**	6.6% [17]	8.6 [12]	6.4% [18]	7.2%

US, United States; UK, United Kingdom; CRS, chronic rhinosinusitis; DM, diabetes mellitus.

The primary objective of this review is to evaluate how the volume of research publications for CRS has changed over the last 45 years and compare the volume of research to asthma and DM. The secondary objective is to evaluate the differences in high-quality publications between each of the chronic diseases. The purpose of this study is to identify potential disparities in research output for CRS, compared to similar chronic diseases, which may be used to improve future research priority setting.

## Methods

The three comparator groups in this review include: CRS, asthma, and DM (both type I and II). Asthma and DM were chosen as comparators due to their similar chronic disease state (often requiring life-long medical therapy) as well as their similar prevalence rates [12-18] and impact on quality of life [9,19,20]. A literature search was performed in December 2014 using Ovid MEDLINE (R) (1970 - December 2014) to define the volume of research publications for CRS, asthma, and DM. The following search terms were used to identify published literature for CRS (terms: ‘chronic’ AND ‘*sinusitis’), asthma (term: ‘asthma’), and DM (terms: ‘diabetes’ AND ‘mellitus’). An unlimited truncation strategy (placement of *) was used to capture all variations of key disease related terms.

After the overall volumes of publications were obtained, limitations for randomized controlled trials (RCTs), systematic reviews (SRs), and meta-analyses (MAs) were applied to separate the overall volume of publications into sub groups. The National Institute for Health Research Economic Evaluation Database (NHS EED) was searched using the same key terms to identify the volume of economic evaluations (EEs) published for each chronic disease. A chi-square (χ2) omnibus test with 2x3 contingency tables was performed to identify significant differences in the proportion of total RCT, SR, MA, and EE publications between CRS, asthma, and DM groups. Significant global tests were followed with bivariate subgroup testing was complete to identify differences between CRS, asthma, and DM groups.

## Results

### Volume of publications

The literature review demonstrated large disparities in volume of publications between CRS, asthma, and DM (Table 2). There were 1,171 articles that overlapped with both CRS and asthma, which represented 15% of the CRS volume and 0.9% of the asthma volume. When evaluating the proportion of research dedicated to high-level evidence (ie. RCTs, SRs, MAs, and EEs), there were differences in the proportions of RCTs, SRs, and MAs published between groups (all <0.018)(Table 2).

**Table 2:** **Publications per chronic disease between 1970 -2014**

**Chronic disease**	**Crude number of publications**	**RCTs (% of total publications)**	**SRs (% of total publications)**	**MAs (% of total publications)**	**EEs (% of total publications)**
**CRS**	7,962	296 (3.7%)	227 (2.9%)	42 (0.5%)	8 (0.1%)
**Asthma**	136,652	8,192 (6.0%)	3,370 (2.5%)	746 (0.5%)	299 (0.2%)
**DM**	337,411	13,633 (4.0%)	7,177 (2.1%)	2,068 (0.6%)	685 (0.2%)
p-value		χ^2^ = 862.4; p < 0.001	χ^2^ = 65.2; p < 0.001	χ^2^ = 8.0; p = 0.018	χ^2^ = 5.6; p = 0.062

CRS, chronic rhinosinusitis; RCTs, randomized controlled trials; SRs, systematic reviews; MAs, meta-analyses; EE, economic evaluations.

The bivariate analysis demonstrated that for the RCTs, there is a significant difference between CRS and asthma (3.7% vs. 6.0%; χ^2^ = 70.6; p < 0.001) as well as asthma and DM (6.0% vs. 4.0%; χ^2^ = 845.8; p < 0.001), but not any significant difference between the CRS and DM groups (3.7% vs. 4.0%; χ^2^ = 2.1; p = 0.148). This indicated that the asthma literature produced the largest proportion of RCTs compared to CRS and DM.

For SRs, there is a significant difference between CRS and asthma groups (2.9% vs. 2.5%; χ^2^ = 4.6; p = 0.032) as well as CRS and DM groups (2.9% vs. 2.1%; χ^2^ = 19.4; p < 0.001) and asthma and DM groups (2.5% vs. 2.1%; χ^2^ = 51.4; p < 0.001). This indicates that the CRS literature contained the largest proportion of SRs followed by asthma and then DM.

For MAs, there is no significant difference between CRS and asthma groups (0.5% vs. 0.5%; χ^2^ = 0.5; p = 0.828), CRS and DM groups (0.5% vs. 0.6%; χ^2^ = 0.9; p = 0.334) but there is a significant difference between asthma and DM groups (0.5% vs. 0.6%; χ2 = 7.4; p = 0.007). This indicates that the DM and CRS literature produced similar proportions of MAs to each other but a larger proportion compared to asthma.

For EEs, there is a significant difference between CRS and asthma groups (0.1% vs. 0.2%; χ2 = 4.9; p = 0.026), as well as between the CRS and DM groups (0.1% vs. 0.2%; χ2 = 4.1; p = 0.043), however no significant difference between the asthma and DM groups (0.2% vs. 0.2%; χ2 = 1.2; p = 0.279). This indicates that the DM and asthma literature produced similar proportions of EEs to each other but a larger proportion compared to CRS.

### Changes in publication volume with time

**Figure 1:**
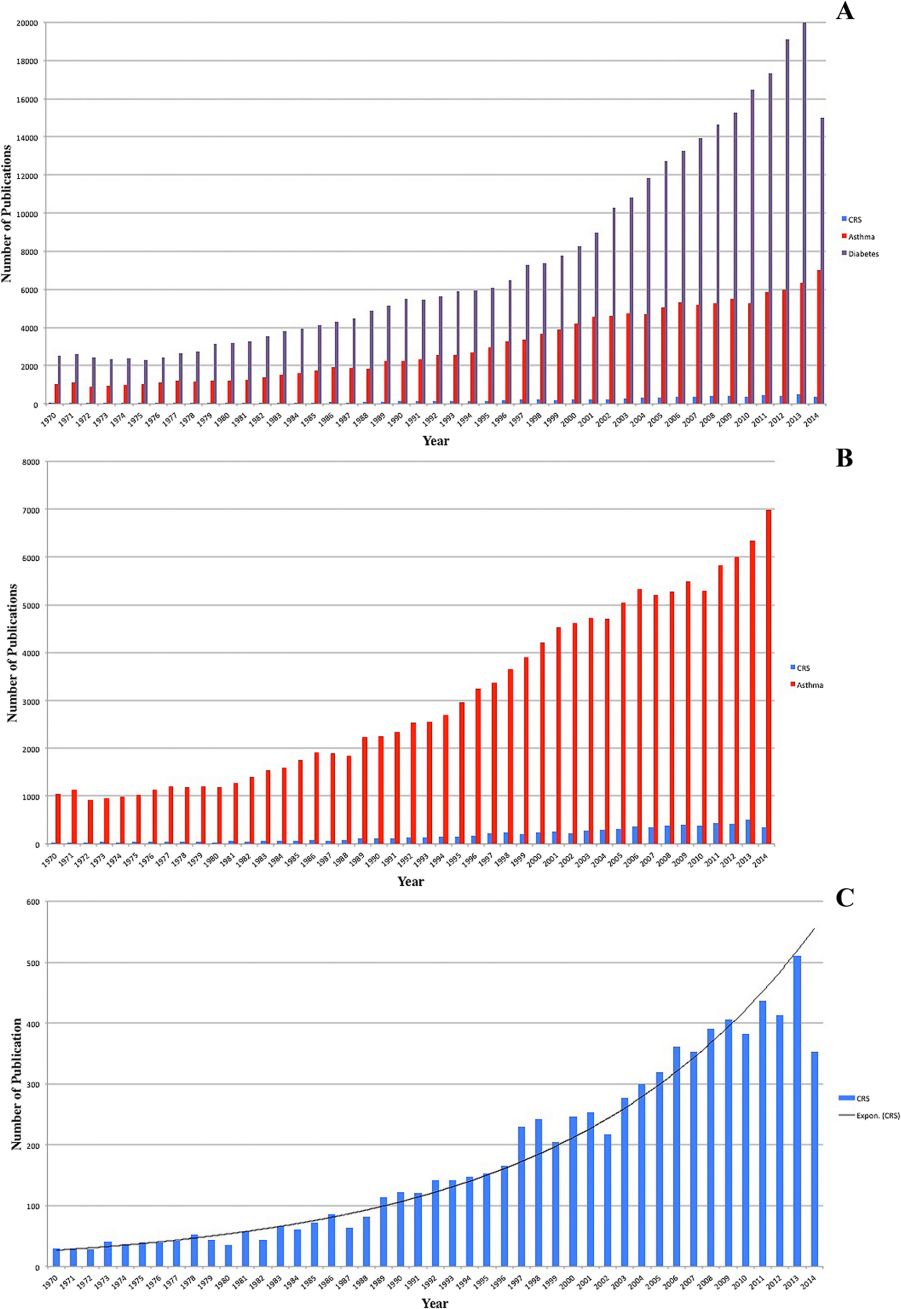
**Publications per year. (A)** DM vs. Asthma vs. CRS; **(B)** Asthma vs. CRS; **(C)** CRS alone.

When evaluating the proportion of research publications per year (between 1970 and 2014), the disparities in volume appear to be consistent over time between CRS, asthma, and DM (Figure 1A and B). There appears to be an exponential increase in the volume of CRS-related publications over the last 15 years (Figure 1C).

## Discussion

Measuring and reporting of medical outcomes using research publications is an important factor to drive quality improvement in health care [21,22]. This review has demonstrated a large disparity in the volume of research publications between CRS, asthma, and DM. Despite having similar prevalence rates, CRS had 6% and 2% of the publication volume compared to asthma and DM, respectively. Although there were minor differences in the proportion of high quality publications (RCTs, SRs, MAs, and EEs), the tremendous gap in overall number of publications is concerning given the fact that CRS results in similar negative impacts on quality of life [9] and economic burden [10,11] to that of asthma and DM (Table 3).

**Table 3:** **Utility, DALY, and economic comparisons between CRS, asthma and DM**

**Chronic disease**	**Health state utility (SF-6D derived)**	**Disability weight**	**Annual direct health care cost**	**Annual indirect cost**
**CRS**	0.65 [9]	Not defined	$3,143/patient [23]	^a^$10,500/patient [10]
**Asthma**	0.70 [19]	^b^0.009 [24]	$4,081/patient [25]	^d^$5,846/patient [25]
		^c^0.027 [24]		
**DM**	0.68 [20]	0.015 [26]	$7,900/patient [27]	$3,920/patient [27]

(Note: all values were adjusted with an inflation calculator to reflect 2014 USD).

DALY, disability adjusted life years; CRS, chronic rhinosinusitis; DM, diabetes mellitus; SF, short-form.

^a^This indirect cost reflects patients with refractory CRS who were recruited from a tertiary level centre and likely over-estimates the mean indirect cost for all patients with CRS.

^b^This is the disability weight for ‘controlled asthma’.

^c^This is the disability weight for ‘partially controlled asthma’.

^d^This indirect cost reflects patients with severe asthma and likely overestimates the mean indirect cost for all patients with asthma.

Research priority setting has focused on burden of disease to justify resource allocation [5,6]. However, without adequate support and awareness, certain medical conditions may be inappropriately under-researched relative to its burden of disease on society. Based on the outcomes from this study, CRS is an example of a highly prevalent chronic condition that is being under-researched given its well-documented negative impacts. This raises the question of: *“Why is CRS being under-researched given its large burden of disease on society?”*.

Although the answer to the above question is likely multi-factorial, one important factor may be the general lack of awareness of CRS in the medical community, as was discussed in a recent article by Tan et al. titled “Chronic Rhinosinusitis: The Unrecognized Epidemic” [28]. This is highlighted by a recent multi-national study intended to quantify the burden of disease for 291 medical conditions around the globe [29]. This study evaluated common chronic diseases such as DM, asthma, coronary artery disease, hypertension, neck pain, osteoarthritis and migraine but failed to include CRS on the list of conditions. The omission of CRS from an international evaluation of burden of disease highlights the lack of awareness for this condition.

Despite CRS failing to be included in two recent multi-national studies quantifying burden of disease [29,30], the high prevalence rate should provide a strong incentive to include CRS in future discussions on research prioritization. The lowest estimate of CRS prevalence is 5%, which came from a 2003 Canadian survey of 73,364 people who were asked “if they have sinusitis diagnosed by a health professional”. Although asking patients for physician diagnosed CRS increases the sensitivity by reducing false positives, it will likely result in an underestimate due to missing people who have not sought medical care for their CRS. This is in contrast to the highest estimate of 12% from the 2014 US National Health Interview Survey (NHIS) which asked patients if they had ‘sinusitis’ rather then asking specifically about whether or not a physician diagnosed their sinusitis. The estimate from the US study will be more specific by increasing the true negative rate, however, it may include false positives such as patients with acute rhinosinusitis or other non-sinus diseases that mimic CRS such as migraines. In 2011 the Global Allergy and Asthma European Network (GA^2^LEN) applied a strict symptom-based approach using the European Position Paper on Rhinosinusitis and nasal Polyps (EP^3^OS) diagnostic criteria to estimate the prevalence of CRS in Europe. This study surveyed 57,128 people from 12 European countries and demonstrated the mean prevalence of CRS was 10.9% (range: 6.9 to 27.1%). Using the strict EP^3^OS symptom-based diagnostic criteria will reduce the false positive rate while maximizing the true negative rate thus increasing both the specificity and sensitivity of the CRS prevalence. Despite the inherent challenges with providing an exact prevalence estimate, the true prevalence rate for CRS in North America and Europe likely falls between 5% and 12% which is similar to asthma and DM (Table 1).

In addition to overall prevalence, the associations between CRS and reduced quality of life and economic burden are other important factors to consider. When comparing health state utility values of patients living with CRS, asthma and DM, the values appear to be similar when using the SF-6D instrument (Table 3). Furthermore, when considering the direct and indirect costs of each of these chronic diseases, DM appears to have larger direct health care costs compared to CRS, however, the indirect cost of refractory CRS is substantially higher then DM. The differences between direct and indirect costs of CRS and DM may reflect increased public awareness of DM resulting in patients seeking more medical therapy. As opposed to CRS, which may not have the same public or physician awareness and result in a substantial proportion of the population failing to receive appropriate medical treatments. The disparities in direct and indirect health care costs between CRS, asthma, and DM warrant future research.

A second potential reason for the disparity in publication volume may include a perception that CRS is a sub-specialty topic, which makes it challenging to publish research in higher impact journals that have a broader audience. This perception may deter basic science, clinical, or pharmacoeconomic researchers from adopting CRS as their academic focus despite tremendous opportunities to provide meaningful contributions. Thirdly, as opposed to CRS, untreated cases of asthma and DM may result in mortality. Although CRS has similar QoL reductions and indirect costs, the difference in mortality risk may have lead researchers to focus on asthma and DM. Lastly, there are fewer rhinologists to perform CRS research compared to the number of general internists, endocrinologists, or pulmonologists, who perform research for asthma and DM. However, given that CRS represents 1-2% of primary care physician visits [23,31], is associated with comorbid allergies in 20 to 60% of cases [32,33], and is commonly associated with asthma outcomes [34,35], CRS research should not only be conducted by rhinologists, but by primary care physicians, allergists, pulmonologists, and general internists. Efforts to increase awareness for the importance of CRS to these medical specialties may help reduce the disparity in publications

This article was intended to evaluate the overall disparities in publication volume between CRS, asthma, and DM. One limitation of this study is the inclusion of one biomedical database (Ovid MEDLINE) which may result in missing certain publications from each category of chronic disease. However, when the EMBASE database was included in the initial search it yielded the same proportion of publication volume, with CRS containing 2% and 6% of the total publication volume as DM and asthma, respectively. Thus, to keep the search strategy and outcomes simple and reproducible, one database was searched to highlight the disparities in publication volume.

## Conclusion

This review has demonstrated a large disparity in the volume of published research for CRS compared to asthma and DM. Given the similarities in prevalence rates, impact on quality of life and economic burden, the relative under supply of research should prompt efforts to improve research prioritization for CRS. Future research should not only begin to elucidate factors contributing to this gap in research volume, but also begin to develop innovative strategies to increase disease awareness for the importance of CRS on population health.
